# How does context influence the delivery of mental health interventions for asylum seekers and refugees in low- and middle-income countries? A qualitative systematic review

**DOI:** 10.1186/s13033-021-00501-y

**Published:** 2021-10-26

**Authors:** Sohail Jannesari, Claudia Lotito, Giulia Turrini, Siân Oram, Corrado Barbui

**Affiliations:** 1 Institute of Psychiatry, Psychology and Neuroscience, Department of Health Services and Population Research, King’s College London, David Goldberg Building, Kings College London, De Crespigny Park, London, SE5 8AF UK; 2 WHO Collaborating Centre for Research and Training in Mental Health and Service Evaluation, Department of Neuroscience, Biomedicine and Movement Sciences, Section of Psychiatry, University of Verona, Piazzale L.A. Scuro, 10, 37134 Verona, Italy

**Keywords:** Interventions, Mental health, Systematic review, Low- and middle-income countries, Context

## Abstract

**Background:**

Low- and middle-income countries (LMICs) host the majority of the world’s refugees. Evidence suggests that refugees and asylum seekers have high mental health needs compared to the host country population. However, they face many social, economic and culture barriers to receiving mental health care and benefitting from mental health interventions. This paper examines how these contextual factors affect the implementation of mental health interventions for refugees and asylum seekers in LMICs.

**Methods:**

We conducted a qualitative systematic review searching 11 databases and 24 relevant government and non-governmental organisation (NGO) websites. We spoke with academic experts and NGO professionals for recommendations, and conducted forwards and backwards citation tracking.

**Results:**

From 2055 records in abstract and title screening, and then 99 in full-text screening, 18 eligible studies were identified. Qualitative thematic synthesis was conducted on eligible papers. Three main thematic clusters were identified around: (1) support during a time of pressure and insecurity, and the need for intervention flexibility through facilitator and participant autonomy; (2) different cultural conceptions of mental health, and how interventions negotiated these differences; and (3) the importance of facilitator skills, knowledge, characteristics and relationships to intervention implementation.

**Conclusion:**

Evidence suggests that intervention coordinators and developers should continue to: (1) think broadly about the range of social influences on mental health, addressing structural issues where possible; (2) offer flexibility with intervention style, content and timings; and (3) encourage building research capacity in LMICs while acknowledging pre-existing mental health knowledge and practice.

**Supplementary Information:**

The online version contains supplementary material available at 10.1186/s13033-021-00501-y.

## Introduction

In 2019, 79.5 million people were forcibly displaced from their homes, the highest number since records began [1]. Among these, 38.8 million sought sanctuary outside their country or territory of origin [1]. These people are likely to have high rates of mental health problems. For example, a recent systematic review [2] synthesised data from 26 studies and 5143 refugees and asylum seekers. The review reported that 32% of participants had depression and 31% had Post Traumatic Stress Disorder (PTSD). It compared these results to the World Mental Health Survey [3], which found rates of 12% and 3.9% respectively for the global population.

Low- and middle-income countries (LMICs) constitute nine of the top ten host countries for refugees and asylum seekers [1]. There is, therefore, a substantial need for refugee and asylum seeker mental health care in LMICs. However, these groups face specific barriers in accessing and benefitting from mental health services in LMICs. Issues include a limited language ability, a lack of awareness of services, discrimination, unstable housing and immigration status [4, 5]. Community sources of mental health support, previously available in people’s home country, may also be inaccessible due to migration [6]. Due to these difficulties in accessing mental health services, mental health interventions with refugees in LMICs are crucial to addressing gaps in mental health support.

Chatterjee et al. [7] argued that LMICs may face particular challenges when implementing mental health interventions. Reasons comprised ‘limited skilled mental health resources, vastly different social and cultural contexts… an already constrained primary care system.. [and] low recognition rates of [Common Mental Disorders] by primary care doctors’ (p39). Jordans et al. [8] similarly claimed that ‘cultural differences, [and] limited human and financial resources’ make the transfer of mental health treatments from HICs to LMICs ‘unfeasible and unrealistic’ (p457). Context, therefore, in the form of culture, society and economics, may be a key consideration in the delivery of mental health interventions in LMICs.

However, context is often loosely defined within health research. For example, as ‘anything external to the intervention that may act as a barrier or facilitator to its implementation’ [14]. In this study, we use Johns’ [9] definition of context from organisational science, adapting it slightly to speak to mental health interventions. Johns defines context as ‘situational opportunities and constraints that affect the occurrence and meaning of organizational behavior as well as functional relationships between variables’ (p386). We define context as the environmental opportunities and constraints that affect the efficacy and meaning of mental health interventions. We follow Johns and other organisational scientists [10, 11] in dividing context into omnibus and discrete context. Omnibus context concerns broad pre-existing conditions and structures such as society’s dominant cultural norms or the biomedical framing of mental health problems; omnibus context is the ‘who, what, when, where and why’ [9]. Within omnibus context, there is discrete, or situational, context. Discrete context takes the intervention environment as dynamic [9] and can be separated into ‘task, social and physical variables’ [9]. This could include the building and lighting a mental health intervention is delivered in.

Contextual factors include concepts that are not easily quantified, for example around culture, religion and social relationships. Qualitative research, therefore, is key to understanding contextual factors in intervention delivery. In particular, Tribe et al. [12] suggests that qualitative methods should be used in future work to understand cultural sensitivity in psychosocial interventions for refugees and asylum seekers.

Qualitative research, in the form of process evaluations, have become a staple of intervention trials [13, 14]. However, Lewin et al. [15] found that process evaluations often do not describe context in any detail. O’Cathain et al.’s review reported that qualitative research during randomised trials focussed on feasibility and acceptability in practice (23%), development (13%) and perceived value (12%) [16]. Few studies (1%) looked at the ‘implementation of the intervention in the real world’ [16]. In exemplifying the last category, the authors refer to a qualitative study on a knee pain advice intervention that provided a ‘model incorporating trial findings and patient preferences into decision-making advice for use in practice’ (p8). Thus, this category referred to studies where research outputs explicitly recommend and outlined changes to delivery.

There is a particular gap in contextual information in mental health intervention studies with refugees and asylum seekers. Uphoff et al. [17] conducted an umbrella review of ‘mental health interventions for involuntary migrants’, which identified 23 relevant systematic review protocols. Of these, only two [18, 19] had qualitative studies in their inclusion criteria with neither, to our knowledge, published at the time of writing. Phillips et al.’s [18] review planned to focus on art therapy and Aslam et al.’s [21] review planned to assess ‘interventions delivered by lay therapists’.

A few authors are trying to address the gaps in knowledge around context and mental health interventions. For example, Chowdhary et al. [20] assessed the feasibility and acceptability of their treatment for depression in ‘seven serial focus group discussions with counsellors’, alongside in-depth interviews of counsellors and patients. These discussions led to a series of changes to the intervention. For instance, they modified their intervention to use ‘appropriate language and metaphors’, ensure the ‘involvement of a significant other in treatment sessions’ and create ‘checklists for use by counsellors’ (p383). Similarly, Kohrt et al. [21] used ethnopsychology, ‘the study of emotions, suffering, the self and social relationships from a cultural perspective’ (p88), to propose mental health interventions for Bhutanese refugees.

Overall, there is a need for a well-defined, qualitative systematic review on how context influences the delivery of mental health interventions for asylum seekers and refugees in LMICs. This study aims to build on and synthesise recent efforts by authors such as Chowdhary et al. [20] to highlight the importance of context in mental health intervention delivery.

## Method

We conducted a qualitative systematic review, adhering to the Preferred Reporting Items for Systematic Reviews and Meta-Analyses checklist and prospectively registered with Prospero (CRD42020176868).

### Search strategy

Online searches were carried between 3 and 6 April 2020 for literature published between 1 January 1967 (the year the UN Protocol Relating to the Status of Refugees was signed) and 3 April 2020. Online databases indexing peer-reviewed literature were MEDLINE, Embase, Embase Classic, Social Policy and Practice, Global Health, PsychInfo, Web of Science, African Journals Online, Intervention and the VHL library (focussing on Latin America). We also searched the grey literature databases Open Grey and the ANLAP HELP library. We contacted authors for further information where data was omitted, required disaggregation or where they had stated that they had conducted qualitative or evaluative work but had not reported on this. Out of five requests, the requested data was provided in three circumstances. Search terms indicative of migration and refugee status (migrant, migrat*, immigrant*, asylum seeker*, refugee*, forced migrants) were combined with terms indicative of psychological illness and interventions (both MeSH terms and text words), qualitative research, and variant spellings and versions of LMIC. Details of the full search strategy are reported in Additional file 1: Appendix S1.

The study authors jointly compiled a list of 25 academic experts from Google searches of recent intervention publications and their knowledge of the field. The list was designed to included academics from a range of countries, with a particular focus on LMICs countries with high asylum seeker and refugee populations such as Bangladesh, Brazil, Colombia, Ethiopia, Iran, Jordan, Lebanon, Pakistan Sudan, Turkey, and Uganda. Each academic was contacted at least three times. They were explicitly asked whether they had conducted any work that might be eligible for our review. A list of NGO experts was similarly compiled and each expert was contacted at least three times. Again, we explicitly asked experts whether there was any work, that they, or their organisation or others in the field, had conducted that may be eligible for our review. Both NGOs and academic experts were also asked for any relevant colleagues we should contact, leading to an element of snowball sampling in compiling the lists. NGO websites were chosen according to the author’s knowledge and a Google search indicating the relevance of published reports.

We received positive responses from five academic experts based in the Netherlands, Egypt, Colombia, the USA and the UK, and eight NGO professionals based in Senegal, Lebanon, the UK, Palestine, Switzerland and the Netherlands, with recommendations on additional potentially relevant papers. We also searched through 21 NGO websites (Additional file 1: Appendix S1). Finally, we conducted forward (using Google Scholar) and backward citation tracking of all the included papers.

### Inclusion criteria

Peer reviewed and non-peer reviewed studies and reports were eligible for inclusion if they: (i) used qualitative methods; (ii) examined non-pharmacological mental health interventions on the second, third and fourth level of the Inter-Agency Standing Committee pyramid [22]. That is, the levels of community/family support, nonspecialised support services, and specialised services respectively. To be considered a mental health intervention, interventions were required to have a mental health outcome as one of their primary outcomes. Mental health outcomes are mental health problems as defined by the International Classification of Diseases [23] or the Diagnostic and Statistical Manual of Mental Disorders [24], or positive psychology traits such as self-control and self-esteem [25]; (iii) targeted and/or included among their intervention a main target population of sanctuary seekers defined as ‘people who have fled their country and [have asked] another country for safety and residence’ [26], this encompasses refugees and asylum seekers as defined by the 1967 UN protocol; OR (iv) included facilitators of any age implementing a mental health intervention while in a LMIC (lay people, NGO staff or mental health practitioners trained in delivering the intervention).

### Exclusion criteria

We excluded studies with only participants in HICs and those who have primarily fled due non-humanitarian reasons. Studies working with internally displaced people and professional mental health staff receiving an intervention, for instance around training, were also excluded. Where studies had mixed findings from eligible and non-eligible populations that could not be disentangled, the study was excluded unless the non-eligible population size was under 10%. There were no study language restrictions. Mental health interventions on the first level of the Inter-Agency Standing Committee pyramid, ‘basic services and security’, were excluded.

### Screening and extraction

A two-stage screening process was carried out on Rayyan by three reviewers (SJ, CL, GT). Reviewers were randomly allocated an equal number of papers for title and abstract screening (685 papers). As a check of reliability, each reviewer independently conducted title and abstract screening on 200 papers belonging to another reviewer. There were no discrepancies. Full text screening was conducted collectively, paper by paper, by all three reviewers at the same time. All papers during full text screening were, therefore, subject to the same screening standards. Disagreements were resolved by discussion and consensus with a fourth member of the team (CB).

A data extraction form was piloted on three papers by each reviewer with post-piloting revisions including the addition of further extraction categories (e.g. duration of intervention and number of sessions). Then, the three reviewers were assigned an equal number of papers and extracted information on bibliographic details, study design and methods, study sample, intervention, contextual factors and findings. Data on context was extracted from the introduction, methods, discussion and other sections as well as the results. The Qualitative Critical Appraisal Skills Programme checklist [27] was used to assess each study on criteria including data collection, ethical considerations and statement of findings. The tool has ten questions that each focused on different methodological aspects of a qualitative study (validity of the study, type of results, if results could help locally). Studies were sorted into high, medium or low-quality based on their overall research value and how well they had accounted for each CASP criteria. Appraisals informed conclusions drawn from the data.

### Analysis

We calculated descriptive statistics on study and intervention characteristics as well as population demographics. Qualitative data was synthesised using an adapted version of Stuart et al.’s method [28], drawing on Braun and Clarke’s [29] thematic analysis guidelines. Papers for coding were split among reviewers equally, who then familiarised themselves with the material and coded a third of their papers.

Reviewers coded participant, facilitator, implementation coordinator, and implementation developer quotes, as well as researcher reflections, interpretations and descriptive contextual information. Often, researchers reflected on their role and insights as implementation coordinators, developers or facilitators. Where relevant, in the coding and results, we have noted the role the researcher takes in their reflections. We have also noted the few cases where implementation coordinators and developers were different people to researchers. This paper partly draws on researcher insights while evaluating or documenting mental health interventions in LMICs. Though  this paper's results and conclusions may be relevant for how researchers conduct their research, this is not a focus or aim of the analysis.

Reviewers then compared codes. This comparison revealed several important topic areas that were only present in the codes of one reviewer. This included codes around academic researcher, intervention coordinator and intervention facilitator biases, cultural differences between participants and researchers, and frustrations between these different parties. A final list of codes was agreed upon based on the discussion. In the next step, reviewers coded and recoded all their data using the final list of codes. When all the data was coded and collated, each reviewer sorted codes to identify and name potential themes. Emerging themes were discussed between reviewers in order to identify shared issues, and to divide them into omnibus and discrete context. The final themes were merged by the lead researcher (SJ), who then produced fundamental thematic clusters.

Themes were shared with four Self-Help Plus (SH+) intervention facilitators in Uganda as well as the facilitator coordinator. SH+ is a novel five session psychosocial intervention developed by the World Health Organisation [30]. After sharing the themes with SH+ facilitators, a 1 h discussion ensued about whether themes reflected their experience. Themes were shared with SH+ facilitators due to author contacts and because there was an ongoing trial in Uganda. These discussions were used to reflect on author interpretation of the themes. Due to time and resource constraints, these were the only intervention that facilitators themes were shared with.

Though the broad thematic framework was endorsed by the SH+ facilitators, the discussion encouraged the authors to revisit the data. Minor points were added to provide more nuance to results, and the focus of a few themes was slightly adjusted. Discussion reflections: (1) clarified that though participants were often disappointed if an intervention offered limited or no material support, they were usually still willing to attend the intervention; (2) suggested that in cases where material support was not provided, facilitators would signpost people as appropriate; (3) emphasised the importance of integrating a mental health intervention with pre-existing charitable programmes; (4) suggested that interventions may begin with facilitators seeking permission from community leaders, even if this is not reported; (5) stated that in working with people with a high level of socioeconomic and psychological needs, facilitators need a broad range of skills beyond what they receive in standard intervention training.

## Results

**Fig. 1:**
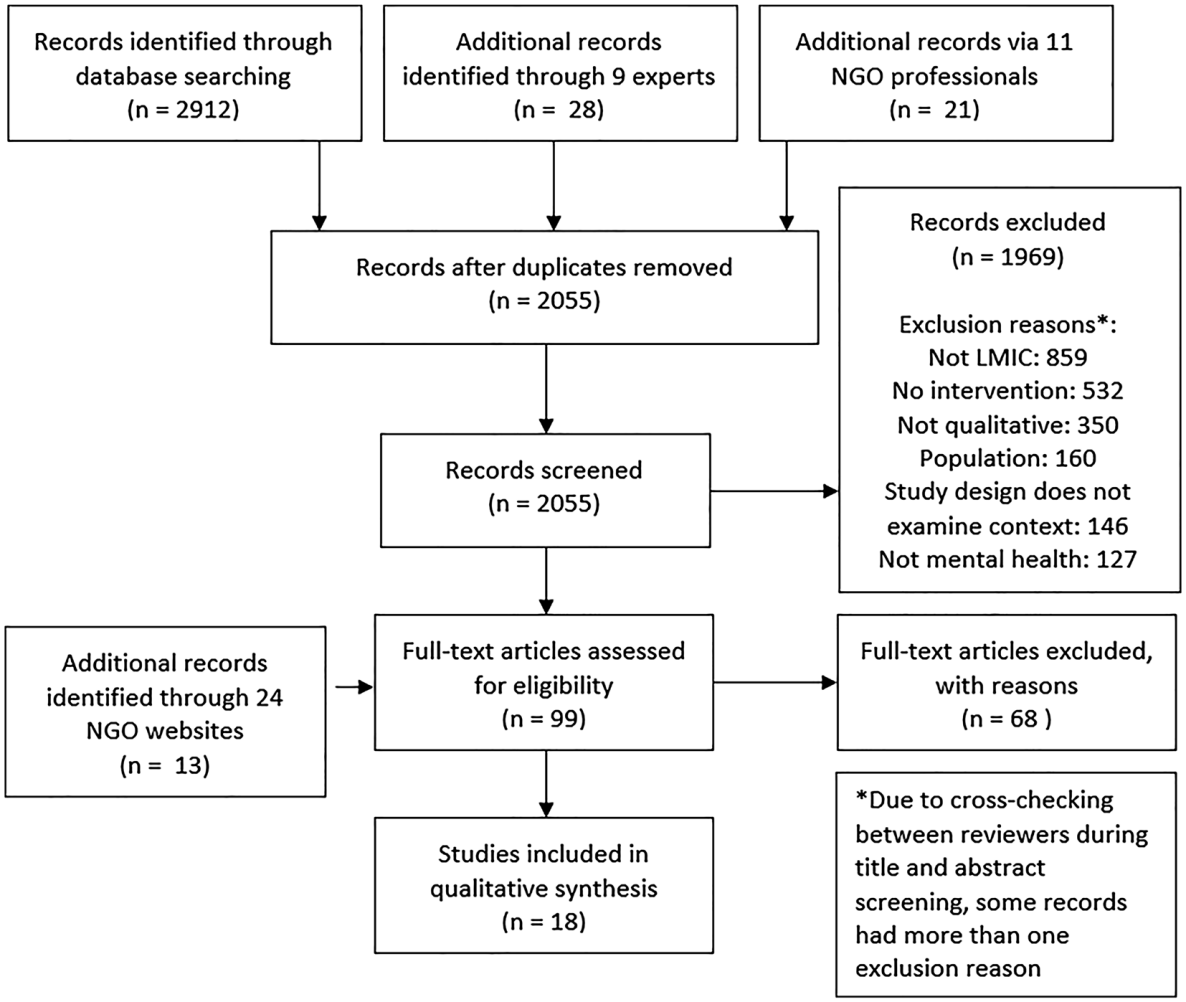
PRISMA flowchart

A total of 2055 unique records were identified from academic and NGO experts, online database searches and tracking of included studies (Fig. 1). Once title and abstract screening was completed, 86 records remained for full-text screening. A further 13 records were added for full-text screening as a result of NGO website searches. Of the 99 articles (or full-texts) then screened, 18 studies were eligible. Key reasons for exclusion were that the studies did not take place in a low- or middle-income country, that they had not examined a mental health intervention, and did not use qualitative methods. All studies took place in the context of piloting, adapting or evaluating an intervention (study information, country, methods and examined interventions are described in Table 1).﻿ Studies were reported in the style of an academic paper (11) or field report (7) [31–48].

**Table 1:** Study aims and intervention

First Author	Year	Study LMIC country	Study aims	Intervention name	Description of intervention	Study method
Aladro	2020	Colombia	What factors influence the implementation of PM+ for Venezuelans	Problem Management Plus (PM+)	PM+ “is composed of four core strategies: stress management (‘Managing Stress’), problem solving (‘Managing Problems’), behavioural activation (‘Get Going, Keep Doing’) and strengthening social support” (p. 87). Includes breathing exercises, task-orientated activities and identifying sources of support	Semi-structured interviews and focus group discussion
Brown et al	2020	Lebanon	To culturally and contextually adapt the EASE program for Palestinian, Lebanese and Syrian adolescents in the north of Lebanon	Early Adolescents Skills for Emotions (EASE)	EASE is focused on “four key empirically supported strategies: understanding my feelings (emotion identification), calming my body (diaphragmatic breathing), changing my actions (behavioral activation), and solving my problems (problem solving). Additionally, three adjunctive caregiver sessions aim to promote positive parenting practices to improve the caregiver–child relationship and enhance confidence when responding to adolescent distress” (p. 3)	Session notes, supervision notes, and a debrief session at the end of the implementation
Burchert et al	2019	Egypt	The adaptation, implementation and scaling-up of Step by Step for Syrians in Germany, Sweden and Egypt	Step by Step (SbS)	SbS is an ‘e-mental health intervention’ consisting of “three core components: the content, the guidance model (e.g., from a human helper) and the delivery system (e.g., web or app). Each of these components can be adjusted and extended to respond to diverse implementation contexts. It is modularized and rooted in evidence-based cognitive behavioral therapy techniques such as behavioural activation, psychoeducation, stress management, increasing social support and relapse prevention” (p. 3)	Interviews and focus group discussions
Chapman & Claessens	2019	Egypt	“To monitor activities and hold focus group discussion with Syrian and Egyptian participants in the activities to collect feedback about the project and re-adjust where required” (p. 9)	Community-based psycho-social support	It is a community-based, non-specialised psychosocial support delivered through local community centres providing psychosocial support sessions, child friendly spaces with special activities, parenting sessions, specialized psychosocial support	Focus group discussions
Greene et al	2019	Tanzania	“To evaluate the implementation of an integrated intimate partner violence and mental health intervention” (p. 6) for Congolese refugee women	Nguvu	“The Nguvu intervention was designed as an 8-session intervention that begins with a single individual session of advocacy counseling followed by six group sessions focused primarily on cognitive processing therapy and a final group session of advocacy counselling” (p. 9)	Interviews
Gürle	2019	Turkey	Exploring the power of drama and art as tools for Syrian children to learn about emotion regulation	Storytelling and image theatre	“The workshop combined both verbal and nonverbal means of expression and primarily employed drama techniques, such as storytelling and image theatre” (p. 166). It included five sessions of two hours	Observation
Hakki	2020	Turkey	To aid Syrian women “to explore changes in their roles and their adversity-activated development after fleeing Syria due to the current conflict” (p. 187)	Theatre of the Oppressed (ToO)	“ToO is theatre. It has three main methods: invisible theatre, forum theatre and image theatre. There were five sessions in total, each session between 90–100 min long, twice a week, and led by one facilitator. The main activities during the sessions were built on a game taken from the ToO approach called ‘What is the story?’ using a shadow theatre technique” (p. 189)	Storytelling methods and group discussion with two open questions
Makhoul et al	2011	Lebanon	To assess the benefits of participating in a community-based research project in a Palestinian refugee camp of Beirut, for Palestinian youth mentors	Youth Mentor Training Programme for Qaderoon	The training for youth mentors began with a three-day workshop. They were then observed during the intensive two-week summer program and provided with feedback. Youth mentors then helped deliver the Qaderoon intervention	In-depth interviews
Murray et al	2018	Ethiopia	To assess implementation aspects of a common elements treatment approach developed among children in three Somali refugee camps on the Ethiopian/Somali border	Common Elements Treatment Approach for youth (CETA)	“CETA is an approach that teaches cognitive-behavioral therapy elements common to evidence-based treatments” (p. 5). It includes elements on ‘engagement’, ‘behvioural activation’, ‘imaginal global exposure’ and ‘cognitive coping’	Semi-structured interviews
Nakkash et al	2019	Lebanon	To describes “the context of Palestinian refugees in Lebanon, the intervention, the process evaluation plan and results” (p. 595)	‘Qaderoon’ (We are Capable)	‘Qaderoon’ (We Are Capable) is a public health intervention. It is “a year-long social skill building intervention for children (11–14 years), their parents and teachers aimed at promoting mental health of refugee children and increasing their attachment to school” (p. 596)	Observation, meetings, discussions and interviews
Rebolledo	2019	Bangladesh	To promote psychosocial wellbeing by increasing sense of identity and community in Rohingya refugees in Bangladesh	Healing Ceremonies Programme	“Healing ceremonies were divided into three sessions (music, art and symbols of strength) to reflect Rohingya culture” (p. 279)	Focus group discussions
Sim et al	2018	Thailand	“To generate evidence around what works to protect Burmese children from the negative effects of forced migration” (p. 7)	Happy Families Program	“It was a group-based parenting and family skills intervention for children aged 8 to 12 and their caregivers. Caregivers and their children participated in parallel group sessions each week, followed by joint activities in which each family practiced the skills that they had learned” (p. 8)	Semi-structured interviews
Sullivan et al	2019	Bangladesh	The 'project utilises Rohingya community health workers... to pilot the use of peer-to-peer teaching of low-cost tools for potential alleviation of mental health complaints' (p.252)	Acupuncture and mindful breathing	It consisted of peer-to-peer teaching of simple relaxation techniques: four acupressure points and breathing exercises. “All six techniques were taught in 90-min group sessions to Rohingya CHWs by explanation, demonstration and practice in same-sex pairs” (p. 254)	Reflective discussion group
Tay et al	2019	Malaysia	“To describe the theoretical underpinnings of Integrative Adapt Therapy, the formulation, development, refinement and cultural adaptation of a treatment manual to guide the intervention, amongst refugees from Myanmar in Malaysia and Bangladesh” (p. 2)	Integrative Adapt Therapy (IAT)	“The Integrative Adapt Therapy integrates universal principles of the Adaptation and Development After Persecution and Trauma model with the particularities of the culture, history of conflict and living context of each refugee community” (p. 1)	Focus group discussions
Tol et al	2011	Uganda	To adapt and pilot a guided, multi-media, self-help intervention, Self-Help Plus in South Sudanese refugees in Uganda	Self-Help Plus (SH+)	“The intervention is based on principles of Acceptance and Commitment Therapy. The SH+ package comprises a pre-recorded audio course and an illustrated self-help manual. The audio-course can be delivered to groups of 20–30 people by lay facilitators trained over a short period. The course consists of five weekly 2-h sessions that include individual exercises and small group discussions (p. 3). In addition, it provides participants with an illustrated manual	Semi-structured interviews
Vijayakumar et al	2017	India	“To assess the effectiveness and acceptability of Contact and Use of Safety Planning Cards in reducing suicidal behaviour among Sri Lankan refugees residing in camps in Tamil Nadu” (p. 590)	Contact and Use of Safety Planning Cards	“The safety planning card consisted of an individualised list of coping strategies containing names and contact numbers of persons in the individual’s immediate family, social circle and health services who could be contacted during a suicidal crisis” (p. 590)	Household survey and focus group discussions
Yassin et al	2018	Lebanon	“To present findings from an evaluation of the refugee camp mental health program conducted among Palestinian Refugees in Lebanon” (p. 2)	Médecins Sans Frontières programme	The program provided free access to mental health care services. “The team provided free access to mental health care services, free access to mental health care services, and provided prescribed medications. Both individual and family psychological consultations were available to all camp residents” (p. 2)	Semi-structured interviews and focus group discussions
Zaghrout-Hodali	2019	Palestine	To describe people’s experiences of eye movement desensitization and reprocessing therapy	Eye Movement Desensitization and Reprocessing (EMDR)	“EMDR is an integrative psychotherapy with protocols that include the use of bilateral stimulation and focus on past trauma, present situations, and future possibilities in enabling the client to reprocess disturbing memories to an adaptive resolution” (p. 248)	Discussions

Studies included in this review reported on a combined total of 677 participants (median = 24, IQR = 48, n = 17), with Chapman and Claessens [35] conducting a case study of four community centres. The smallest study worked with only 3 people [43] and the largest with around 150 [48]. Around 62% of these participants were women (392, n = 13). Most studies worked with only adults (11), though a few worked with mixed populations (3) and children (4). Syrian (5 studies) and Palestinian (5 studies) were the most common participant nationalities.

Research occurred in a range of countries including Lebanon (4 studies), Turkey (2), Egypt (2) and Uganda (1). Lead authors were based in the USA (4 lead authors), Lebanon (3), the UK (2) and Turkey (2). Almost all researchers used either interviews (9 studies) or focus groups (7). A few studies used less formal methods such as discussion and note-taking (3).

Among the included studies, 13 of 18 interventions used and adapted work by lead authors based in universities or NGOs in Western (high-income, majority white) countries, most frequently the US (7 interventions). Though Cognitive Behavioural Therapy was a common basis for intervention development (5), many interventions drew on non-Western practices and philosophies. For instance, Buddhist ideas around mindfulness informed the SH+ intervention [53] and Pranayama Yoga’s breathing exercises the intervention in Sullivan and colleagues' work [37]. Studies were generally of medium quality with almost all reporting clear aims, design and data collection strategies. See Additional file 2: Appendix S2 for a full study by study quality appraisal table.

Most interventions (eleven) were not based on an explicit theoretical or rigorous trial evidence. Rather, they were created through NGO needs assessments, practices, and experience; qualitative interviews with practitioners and people with lived experience of a particular issue; or drew on pre-existing cultural practices around mental health (not necessarily from local cultures). Seven interventions were explicitly created around a theoretical framework. For example, Tay and colleagues [47] developed Integrative Adaptive Therapy by drawing on the Adaptation and Development After Persecution and Trauma (ADAPT) framework. Silove [49], the academic who developed ADAPT, suggests it offers ‘a unifying, conceptual framework’ and argues that the each of its pillars is supported by over a decade of empirical studies.

**Fig. 2:**
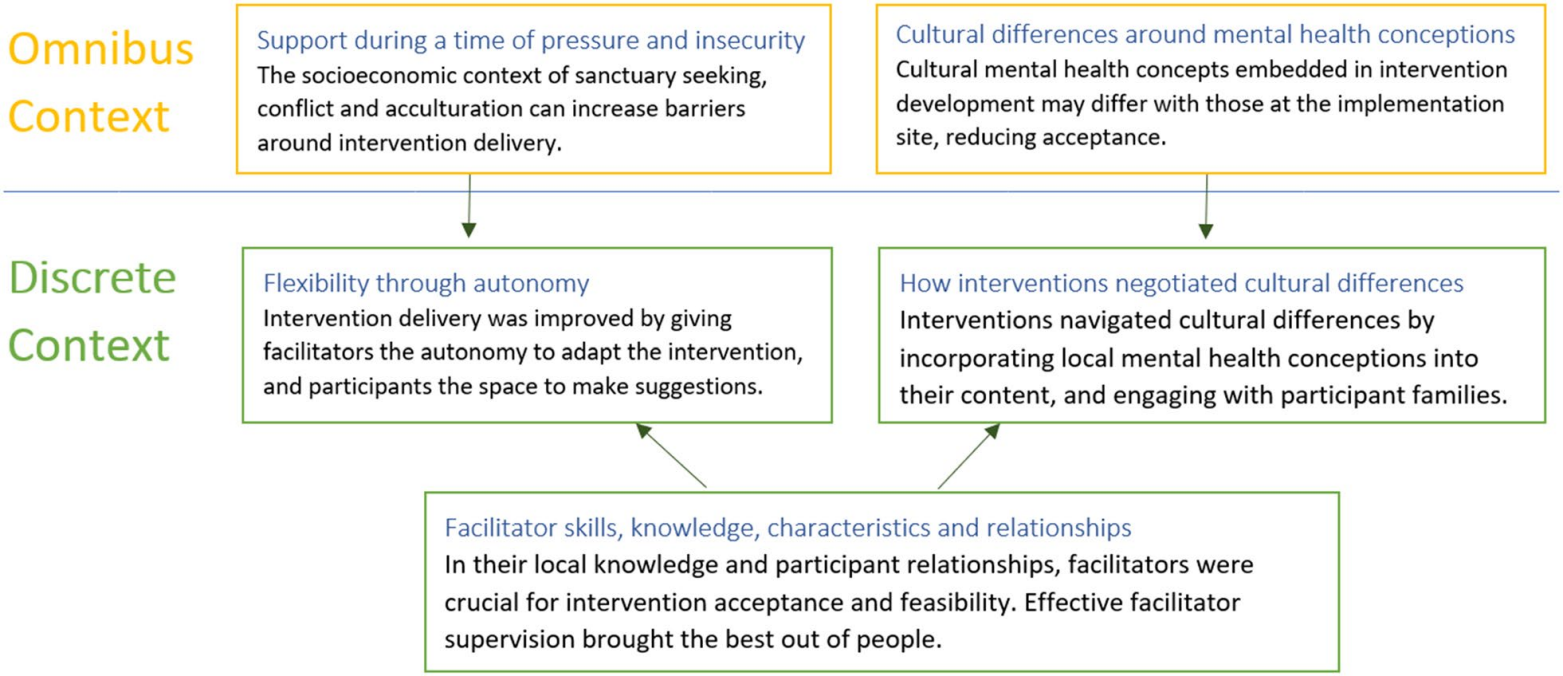
A map of themes by omnibus and discrete context

Thematic analysis produced three thematic clusters (Fig. 2)﻿. These were divided into themes on the omnibus contextual level, around the broad context in which interventions were implemented, and on the discrete contextual level, around contextual factors particular to the intervention implementation. The thematic clusters were: (1) support during a time of pressure and insecurity (omnibus context), and flexibility through autonomy (discrete context); (2) cultural differences around mental health conceptions (omnibus context), and how interventions negotiated cultural differences (discrete context); and (3) facilitator skills, knowledge, characteristics and relationships (discrete context).

Support during a time of pressure and insecurity speaks to the insecure and socioeconomically precarious environment many interventions were implemented in. Flexibility through autonomy shows how, within these environments, interventions needed to be flexible and adapt to their participants’ changing circumstances. This could partly be achieved by giving participants more control over the intervention. Cultural differences around mental health conceptions referred to the cultural context of intervention development and delivery, typically created by Western-based researchers for non-Western participants in non-Western countries. It suggests that this may link to cultural misunderstandings around conceptions of mental health and, potentially, reduced intervention acceptance. Within the theme on how interventions negotiated cultural differences, there is a description of how intervention coordinators tried to resolve these misunderstandings by building relationships with participants, their families and their communities, and by making ad hoc cultural adaptations to their intervention. Facilitator skills, knowledge, characteristics and relationships were identified as an underlying discrete theme both positively and negatively affecting participant autonomy as well as facilitating the intervention coordinator’s cultural communication.

### Cluster 1 omnibus theme: support during a time of pressure and insecurity

Many intervention participants had left their country under difficult circumstances and were living in temporary and precarious housing with limited economic prospects. Some people were still living in the backdrop of violent conflict or humanitarian crises. Ensuring that interventions contributed practically to wider economic and social needs was important to acceptability. Creating flexibility schedules that recognised people’s insecure and stressful lives was crucial to feasibility.

Most sanctuary seeker participants reported that they sought pathways out of their precarious economic situation, and towards security. Accordingly, mental health interventions providing work opportunities and skills for adults, or education for children, were well-received.

*Incentives to join the Qaderoon intervention: a variety of “new opportunities” for the mentors themselves (15/18), such as a stable income for the duration of the project and an opportunity to be involved in social work.—Makhoul et al., p918*

*The life skills sessions have evolved to also include computer skills (introduction to Microsoft office) and English language classes, based upon requests and needs of the Syrian children and youth.—Chapman and Claessens, p7*

Some participants across eligible studies, therefore, reported having been disappointed when an intervention did not offer to assist with basic goods and services. Yet, authors reported that this neither reduced attendance nor the continued hope of practical support.

*[Participants wished] that they were provided with other services outside the scope of CETA (e.g. requested materials goods like blankets and sleeping maps, assistance with relocation).— Murray et al., p10*

*Although the nature of the program, including the lack of material goods and services provided, was explained… some participants experienced disappointment when they attended sessions and were not provided with material goods—Tol et al., p9*

Though people continued to attend interventions despite a lack of goods and services, employment was a barrier for few participants. They described how they worked long hours and had limited time to engage in interventions, which were usually during the day. They occasionally had to cancel attendance because of a last-minute job offer or due to their variable work schedule.

*During the five sessions there was some degree of fluctuation in participation levels due to Ramadan and seasonal labour demands—Gürle, p5*

*One challenge raised by all counsellors relates to scheduling difficulty given that the majority of the Rohingya refugees in Malaysia have full time employment and are only available during evening hours.—Tay et al.*

Participants also reported a plethora of other everyday tasks that could prevent them from attending intervention sessions, including cooking, cleaning, school and childcare. Activities and practice outside of the intervention were, therefore, highlighted by participants as very difficult to manage.

*The reasons for absence from sessions included health problems, parenting and childcare, as well as prior engagements (e.g., funerals, school, work/chores).—Greene *et al*., p10*

*5 participants reported difficulties in completing the assigned homework following the sessions: “I found it difficult to practice the tasks given to me given my busy schedule, but I tried to do my best”—Tay et al.*

Contextual information from researchers, as well as participant descriptions, indicated that people typically lived in socially insecure situations. They felt little sense of belonging and experienced prejudice and discrimination from the host community. Reassuring participants that interventions would try not to reproduce these issues helped with trust and acceptance.

*[Facilitators] dispelled concerns of volunteers reporting Venezuelans to authorities or armed groups. This, they argued, was especially important at a time when there was news of Venezuelans being killed by armed groups in Saravena—Aladro, p160*

Insecurity went beyond the personal circumstances of the participants, also including the social and political environment in which people lived. Interventions often took place in areas in or close to armed conflict, or in large scale humanitarian crises. Consequently, there were cases where the intervention had to be postponed, stopped or certain elements reduced (e.g. facilitator supervision).

*Israel attacked the Gaza strip for a period of three consecutive weeks resulting in a high number of Palestinian civilian casualties and tremendous destruction of civilian property and infrastructure… we decided to halt all ‘usual’ activities—Nakkash et al., p604.*

### Cluster 1 discrete themes: flexibility through autonomy

Participant and facilitator autonomy was key to achieving the flexibility needed to accommodate the everyday pressures and insecurity participants were living through, as well as their cultural values. Facilitators needed the freedom to be able to adapt the content, style, and timings of interventions to match the specific demographics and needs of their cohort. Being able to draw on a range of communication mediums was important to this. Equally, participants needed to be given the opportunity to suggest improvements around implementation and timings. For the seven interventions based on theoretical frameworks and rigorous trialling evidence, flexibility was much more difficult, particularly in terms of content. For these interventions, fidelity was key to effectiveness. However, even these interventions reported that flexibility around intervention scheduling and delivery medium was useful.

Many facilitators underscored the importance of autonomy to alter the intervention style and even content. For example, choosing to deliver to groups or individuals, or changing elements of the content, including in response to cultural sensitivities such as those relating to gender.

*[Services] may include flexible one-to-one sessions for those that cannot attend group sessions, or online or telephone options.—Brown *et al*., p14*

*[We removed] the movement and using the body in theatre elements. The participants had refused this element of the sessions because they considered it culturally inappropriate for women of their age.—Hakki, p190*

Most participants and facilitators felt that being flexible with the time, length and date of an intervention was imperative to its successful implementation. Given the unpredictable lives many participants lived, it was important to allow those who had missed sessions time to catch up, or to offer the chance to reschedule.

*There were challenges where children or caregivers had missed a session and attended a later one, because limited time was available to cover the missed material—Brown et al., p12*

*The opportunity to reschedule or change the setting of the PM*+ *sessions were identified as useful strategies contributing to beneficiaries’ autonomy—Aladro, p182*

According to most facilitators and participants, using a plurality of ways to communicate with participants was essential with some participants having low literacy and education levels. Communication methods included the use of images, role play, audio and demonstration.

*‘[Community Health Workers] reported that using pictures and demonstration were effective teaching methods’—Sullivan *et al*., p257*

*Caregiver and adolescent low education and low literacy were common challenges… Facilitators often had to offer adaptations as required for literacy challenges (e.g. options of drawing).—Brown et al., p12*

Nonetheless, simple handbooks and manuals were also valuable in helping participants understand the mental health intervention. They gave people a chance to refer back to concepts outside of intervention sessions. Participants preferred simple supplementary materials as they were more understandable.

*‘I really liked the toolkit which I found very creative and effective in getting across the essence of IAT’—Counsellor in Tay *et al*.*

*‘The majority of participants considered the SH*+ *materials such as the illustrated manual and the worksheets useful… Even those who were unable to read Juba Arabic indicated that they understood the meaning from the illustrations in the manual and enjoyed looking at the illustrations, particularly after attending the SH*+ *sessions.’—Tol et al., p9*

Facilitating space for participants to make suggestions on intervention delivery helped the contextual adaptation and implementation of interventions.

*Participants... suggested measures to improve technical literacy (e.g., training courses or multimedia tutorials)—Burchert *et al*., p7*

*Participants suggested that improving communication and organization would also improve safety because women would be aware, in advance, of the exact time and date of sessions.—Greene et al., p10*

### Cluster 2: cultural differences around mental health conceptions

On the level of omnibus context, the culture in which interventions were developed were usually different to participant culture during implementation. Though intervention developers culturally adapted their interventions, this difference was still relevant and manifested in cultural misunderstandings, and potentially resulted in reduced intervention acceptance. Using mental health concepts outside of biomedical framings, for instance around religion and wellbeing, helped bridge this cultural gap.

Despite often rigorous cultural adaptation processes, participants in most studies still reported a lack of familiarity with some mental health concepts and treatments. Hence, interventions were not intuitively understood and acceptance could take time, often increasing as participants experienced improvements in their wellbeing.

*Many did not take the breathing exercises seriously at the beginning, reporting that they thought it took too long or that it was difficult to concentrate on… difficulties with the breathing exercise might be due to… [it] not being commonly used in this setting.—Aladro, p121*

*Stigma and hesitance turned into acceptance as many individuals saw changes in themselves and others who were receiving treatment.—Yassin *et al*., 394*

Interventions did not always align with participant cultural mental health framings. A few participants interpreted mental health via a religious lens and hoped that research would strengthen their religious community. This understanding was rarely reflected in interventions.

*[Engagement may have been impacted by] clients’ religious and cultural views… general belief in the camps was that mental health issues came from a higher religious power… the most common practice to treat mental illness is to have the afflicted parties read religious scripture… making counselling a somewhat foreign form of treatment—Murray et al., p10*

Many participants and intervention coordinators were wary of the cultural stigma from participant communities associated with biomedical mental health framings.

*When the program began, community members were hesitant to seek treatment because fear of stigma was prominent in the community since going to the center was associated with “being crazy”—Yassin et al., p390-394*

Accordingly, many interventions presented their work as a wellbeing and community project, increasing acceptance and attendance.

*‘[The intervention goal is to] strengthen positive coping mechanisms and resilient responses to foster a more cohesive community’—Rebolledo, p279*

Wellbeing was viewed by participants and facilitators on a communal level, and they were motivated to engage with an intervention if it benefitted the community. Relatedly, participants were proactive in recommending that the intervention be expanded to a wider range of people.

*“What I liked about the project is that it involves helping the children in the camp. I passed through problems and situations when I was young and I don’t want my little brother or cousin or neighbor to commit the same mistakes I did previously.”—Participant in Makhoul et al., p918-919*

*Two participants suggested that IAT should be rolled out across all refugee communities in Malaysia and elsewhere: “When I shared my experience with my friends in Bangladesh about this program they were interested and wanted to apply”.—Tay et al.*

### Cluster 2 discrete themes: how interventions negotiated cultural differences

To navigate the cultural misunderstandings around mental health arising from the omnibus context, intervention coordinators adopted practices and delivery sites relevant to people’s culture. These dynamic changes, encompassed within discrete context, helped intervention acceptance and participation. This process was assisted by the development of trusting relationships with participants and securing acceptance not only from participants, but also participant families.

Many facilitators, intervention coordinators and researchers found that when interventions incorporated mental health terms and practices appropriate to participants’ religion and culture, they were readily accepted. For instance, a few interventions used a religious site as their delivery setting, included local mental health treatment practices and used locally understood concepts to speak about mental health.

*Cultural and religious concepts such as metta or “loving- kindness,” also discovered through the course of qualitative research, were incorporated in the intervention to facilitate discussion around the negative effects of harsh punishment in a way that resonated with participants’ existing beliefs.—Sim et al., p8*

*The two categories of resilience and response were merged together and any negative qualitative feedback from participants was labelled ‘suffering’, to make it clearer for participants and more culturally appropriate.—Hakki, p191*

A few researchers reported that organically developing relationships between participants helped create an atmosphere of trust and encourage the acceptance of new, culturally unfamiliar ideas.

*The small group of women were from different economic and social backgrounds, but they shared that they were like queens in their houses… There was a fast connection between them from the first minute of session one, as they were prepared for the sessions to start and had some pre-conflict experiences in common.—Hakki, p192*

Participants reported that individual-level acceptance of an intervention’s conceptualisation of mental health was insufficient as negative attitudes among the family could pose significant barriers to engagement. Participants and intervention coordinators stated these barriers were typically only overcome when the family and community saw a direct benefit.

*A few patients reported that family members still did not accept their illness, sometimes criticizing them, which forced them to seek therapy in secret to avoid being labelled [sic] as a mental health patient.—Yassin et al., p394*

*‘Family members (mothers or sisters) explained that because of Qaderoon, [Youth mentor] “relationship with their families strengthened” immensely… [they] now enjoyed spending time with them at home [and] cared about their mothers’ and siblings’ wellbeing’—Makhoul et al., p922*

### Cluster 3 discrete themes: facilitator skills, knowledge, characteristics and relationships

In almost all review studies, the local knowledge and positive participant relationships with facilitators played an irreplaceable role in intervention acceptance and feasibility. Intervention coordinators encouraged facilitator contributions through effective supervision and training, and creating an intervention with a positive social contribution.

Most local facilitators, intervention coordinators and study researchers felt that facilitators formed a crucial link and point of contact between foreign researchers and participants. They were typically well-respected, serving as a source of advice for participants while improving acceptability.

*The CVs (community volunteers) who served as an important point of contact with the community were well received, seen as individuals who were both supportive and encouraging and who gave useful advice. This motivated the intervention participants into engaging in behaviours that were positive and not destructive—Vijayakumar et al., p593*

*[Community Health Workers] are often the first responders in emergencies, have gained the respect and trust of the Rohingya community… it was recognised that this large cohort of respected community members could be an appropriate conduit to introduce innovative self-help tools for mental wellbeing—Sullivan et al., p1*

Researchers reported that facilitators often helped explain the intervention to participants, and were vital in improving accessibility.

*When confusion arose during a session, facilitators would pause the recording and offer a clarifying explanation.—Tol *et al*., p9*

*Many specifically acknowledged the role of the facilitators or “teachers” in providing guidance and knowledge, suggesting that respect for the facilitators played an important role in encouraging uptake.—Sim et al., p20*

A few intervention coordinators and facilitators stated that character traits, such as self-confidence and social skills, were useful in supporting the successful delivery of interventions. Those who were not strong in these areas gained in confidence through the process of delivering the intervention where appropriately supervised and supported. This character-building increased the commitment of a few facilitators.

*A PM*+ *volunteer should have strong communication skills, feel comfortable and inclined to speak in public, should feel at ease approaching different people about psychosocial support, and be able to manage their emotional reactions in the face of difficult circumstances—Aladro, p164*

*“I noticed some changes in me after delivering IAT to so many participants. For example, I can understand my own struggles much more deeply. This insight really helped me put everything into perspective and now it all makes sense to me why I am feeling this way”.—Counsellor/Facilitator in Tay et al.*

Facilitators also reported feeling more dedicated and invested towards the intervention when they could see a benefit to their community and felt part of a positive social cause.

*“I liked the children more than anything in the project. I liked the children a lot. I felt that I was benefiting someone. Really for the first time I feel that I am doing something for someone, that is someone in need. I’m doing something good for the camp.—Participant in Makhoul et al., p919*

Researchers contended that adequate and continued training for facilitators, including and beyond intervention delivery, was critical to providing them the skills and tools they needed to cope and thrive in the intervention context. An experienced supervisor was also helpful.

*This supports… findings that externalizing problems are salient in this population, and highlights the importance of training facilitators in additional behavioral management strategies in Lebanon.—Brown et al., p6*

*Mentors were then observed during the intensive two-week summer program and provided with feedback… as well as a follow-up one-day workshop prior to the initiation of the rest of the intervention—Makhoul et al., p917*

Despite facilitators delivering the intervention and having a wealth of relevant experience, few intervention coordinators reported allowing space for facilitators to make suggestions to intervention changes. Tol et al. were a significant exception.

*During and after supervision, facilitators provided additional suggestions for changes separate to the process evaluation including, removal of worksheets (due to literacy concerns), simplifying the audio script, reducing the length of sessions and some exercises, adding audio cues to signal when a facilitator had to do an activity, and showing relevant illustrations from the SH*+ *manual to support understanding of key concepts—Tol et al., p6*

## Discussion

This systematic review generated three thematic clusters on the influence of context in the delivery of mental health interventions for sanctuary seekers in LMICs. These were on the importance of: (1) using culturally relevant mental health conceptions and framings, and negotiating these cultural differences; (2) intervention flexibility to adapt to the insecure circumstances participants lived in; and (3) facilitator local knowledge and participant relationships to feasibility and acceptance.

Omnibus themes focussed on how cultural differences and socioeconomic insecurities may affect the delivery of mental health interventions in LMICs. Though these themes are broadly applicable to most people in LMICs, results suggest that they may have specific relevance in sanctuary seeking populations. Economically, interventions must consider the limited access sanctuary seekers have to economic opportunities, such as employment, even when compared to the local LMIC populations. Sanctuary seekers are also exposed to social insecurities around discrimination and belonging. Culturally, interventions must adapt both to the receiving country cultural context and sanctuary seeker cultures. The latter is crucial as acculturation is a key postmigration mental health stressor [50]. Facilitators who received adequate training, supervision and lived experience could help guide intervention coordinators through these cultural and socioeconomic challenges.

### The cultural context of delivery

Though most included studies worked to culturally adapt interventions developed in Western settings, some issues persisted around cultural acceptability and language. Adaptation did not render the omnibus context of culture null. This could suggest that not all mental health concepts are universal and that there may be limits to the cultural adaptation process. We found that intervention acceptance typically increased when culturally specific mental health concepts were incorporated. This chimes with recent reviews of mental health research in humanitarian settings [51, 52] contending that mental health research veers too far towards an etic perspective, and calling for a more combined emic-etic approach.

Acknowledging the influence of different cultures in Western conceived interventions may help the cultural adaptation process. Understanding the cultural background, history and identity of a theory will help maintain its most crucial elements, and emphasising this may help acceptance in certain populations. Moreover, the Inter-Agency Standing Committee guidelines [22] suggests that a recognition of culture can improve wellbeing. For example, a few studies included in this review [34, 37, 40, 46] state that interventions draw on the practice of “mindfulness”. However, none refer to its Buddhist or Hindu religious origins [53], its colonial history and Western appropriation [54], and the links between ‘secular mindfulness’, neoliberalism and white supremacy [55].

Alongside this acknowledgement, it would be useful to increase the recognition of pre-existing LMIC mental health practice. There is a plethora of home country mental health traditions that can be adapted for use with migrants [56]. Some of these could potentially be scaled-up to meet the needs of sanctuary seekers in LMICs. Methods reflecting the heritage of black and brown people are already being adapted in Western countries for use in the diaspora [57]. We advocate for increased capacity building efforts in LMICs to support the creation of LMIC-conceived interventions. We welcome developments in projects like the African Mental Health Research Initiative [67], which provides support for four universities in LMICs to develop mental health research programmes and leadership.

### The socioeconomic context of delivery

Interventions in resource-limited settings needed to be delivered flexibly, recognising that participants may struggle to attend all sessions. Though studies were generally flexible with timings, almost all of them required participants to physically attend. Burchert et al.’s [33] smartphone app intervention was the main exception, however they experienced issues around technological literacy and access. In the context of recent epidemics and the current COVID-19 pandemic, it is imperative to address this gap in practice. We echo Ruzek and Yeager’s [58] calls for ‘simple text messaging interventions’. Wang et al. [59] provide another, potentially low-cost option, offering participants in China MP3 players loaded with an audio of the intervention. Moves to deliver interventions remotely or digitally, however, should not take away from the integral role of facilitators whose local links and relationships will still be essential for acceptance and understanding.

Relatedly, interventions could be designed with participant choice and flexibility in mind, with findings suggesting that facilitator alterations to style and content of intervention delivery improved acceptance. Facilitators and participants could be provided a choice of intervention activities. Mobile mental health apps like Headspace, for example, offers its users a selection of programmes to choose from each day. Evidence suggests that such mental health apps can improve wellbeing [60]. Including a choice of options supports participant and facilitator autonomy whilst ensuring that fidelity can be, to some extent, maintained for more rigid interventions. Choice in the delivery and content of mental health interventions may be particularly important in the sanctuary seeking context. This is because a lack of perceived control has been linked to mental health problems during the asylum process [61].

Participant social issues could be addressed by embedding interventions in larger institutions. In Aladro’s [31] work with the Red Cross, for example, intervention delivery was supplement with access to assistance from elsewhere in the organisation. To an extent, our findings reflect the move in recent decades towards psychosocial support [62]. However, we found that people sought support over and above this. They wanted to improve their long-term educational and economic prospects, and strengthen their social environment. Intervention developers and coordinators need to: think broadly about the range of social influences on mental health; consult with the target population in designing the intervention; and engage with external organisations and services in working to support needs.

Mental health interventions should also target omnibus contextual factors. They could try to change the structure and aims of mental health services, and think about how these can challenge fundamental socioeconomic forces. We echo Horn’s [63] call for mental health services to take a more activist position. Participatory Action Research could be a useful approach for intervention developers as it encourages participants to identify structures of oppression and take action to address them [64, 65]. Intervention coordinators should work with participants to think carefully about the appropriate context and safety precautions around social action, facilitating an informed participant decision. Sanctuary seekers may have precarious status and could risk deportation if they are arrested at a protest.

### The importance of facilitators

Facilitators were crucial to forming links with the community, improving understanding, recruitment and acceptance at every level. They were the most influential actors in the realm of discrete context. Facilitators often aspired to help their community and it may have been useful to collaborate with them on sustainability. Facilitator roles were typically so substantial, and their knowledge so deep, that they could have acted as co-intervention developers and coordinators. As some facilitators reported wellbeing improvements during their role, future interventions may want to follow Makhoul et al.’s [38] example of assessing benefits—and potential harms—to facilitators, and factoring it into their design and outcomes.

### Strengths and limitations

The review used a comprehensive search strategy, including searches of databases indexing grey literature and literature primarily from LMIC settings, and expert recommendations. However, searches were only conducted in English, limiting access to substantial LMIC databases, for instance in China. Our review benefitted from sharing themes with intervention facilitators in LMICs, ensuring that themes reflected people’s lived experience and replicating an emerging practice in the field [28]. Some studies were primarily focussed on intervention efficacy and only briefly mentioned contextual factors. In these cases, we asked for, and in several circumstances received, qualitative research reports, summaries and further information. Few studies discussed ethical issues or the relationship between participants, and facilitators/intervention coordinators in any meaningful way. This is worrying given the power imbalances and potential for exploitation linked with migration research [63]. Studies also were poor at describing their analysis process, a common criticism of qualitative research [66]. The qualitative methodology used in this paper limits the generalisability of the findings. However, we hope that the insights drawn from our interpretation of these specific papers is useful for future intervention developers working in LMICs.

## Conclusion

To maximise acceptance, mental health interventions for sanctuary seekers in LMICs must work to improve people’s social environment. In the context of social and economic insecurity and the COVID-19 pandemic, interventions also need to be flexible in their means and mediums of delivery. Flexibility should also encompass responding to feedback from participants and facilitators. Ultimately, we emphasise the need for capacity building efforts in LMICs and recognition of pre-existing home country mental health practices to support the creation of more culturally acceptable interventions.

## Supplementary Information

**Additional file 1: Appendix S1.** Search Strategy. 

**Additional file 2: Appendix S2.** Quality Appraisal. 

## Data Availability

Review primarily used pre-existing data available on academic databases. The extra data sent by authors is not ours to share.

## Abbreviations

NGO: Non-governmental organisation
LMICs: Low- and middle-income countries
