# Supplementary material for: How does context influence the delivery of mental health interventions for asylum seekers and refugees in low- and middle-income countries? A qualitative systematic review
Source: Int J Ment Health Syst. 2021 Oct 26;15:80. doi: 10.1186/s13033-021-00501-y (PMC8546788; doi:10.1186/s13033-021-00501-y)
Supplement: Supplementary file 1 — Additional file 1: Appendix S1. Search Strategy. [file 13033_2021_501_MOESM1_ESM.docx]

**Search Strategy by database**

**Ovid MEDLINE, Embase, Embase Classic, Social Policy and Practice, Global Health, PsychInfo -1919 results**

| 1. (asylum seek$ or refugee$ or migrant$ or immigrant$ or forcibly displaced or irregular migrants or undocumented migrants or forced migrant or externally displaced or refugee camp).mp. |  |
| --- | --- |
| 2. refugees/ or human migration/ or "emigration and immigration"/ |  |
| 3. exp mental disorders/ or exp Mental Health/ |  |
| 4. (mental health or ((mental$ or psychologic$ or psychiatr$) adj2 (health or disorder$ or disease$ or deficien$ or illness$ or problem$))).mp. |  |
| 5. exp Psychology, Positive/ or (flourish$ or positive psychology or health promotion or positive mental health or subjective well-being or subjective well being or subjective wellbeing or mental well-being or mental well being or mental wellbeing).mp. or resilience, psychological/cl, es or resilience.mp. or Adaptation, Psychological/cl, ed, es |  |
| 6. Depressive Disorder/co, di, dh, dt, ec, ep, eh, et, hi, mo, nu, pc, px, rh, sn, su, th or anxiety disorders/co, di, dh, dt, ec, ep, eh, et, hi, mo, nu, pc, px, rh, sn, su, th or PTSD/co, di, dh, dt, ec, ep, eh, et, hi, mo, nu, pc, px, rh, sn, su, th |  |
| 7. cross-cultural comparison/ or cultural characteristics/ or cultural diversity/ or ethnology/ or Qualitative research/ or qualitative.mp. or ethnological research.mp. or (cultural adaptation or crosscultural adaptation or cross-cultural adaptation).mp. or ((purpos$ adj4 sampl$) or (focus adj group$)).mp. or 'observational method$'.mp. or interview.mp. or content analysis.mp. or ((discourse$ or discurs$) adj3 analys?s).mp. or 'narrative analys?s'.mp. or (grounded adj (theor$ or analys?s)).mp. or 'action research'.mp. or (account or accounts or unstructured or openended or open ended or narrative$).mp. or process evaluation.mp. or (feasibility or acceptability).mp. |  |
| 8. Internet-Based Intervention/ or (service$ or delivery or provision or model or programme or program or treat or therapy or psychother$ or psychosocial or therapy or intervent$ or counsel or support$ or intervention).mp. |  |
| 9. exp Global Health/ or exp Developing Countries/ or (developing countr$ or developing countr$ or developing nation$).mp. or (low- and middle-income countr$).mp. or "low and middle income countr$".mp. or "low- and middle-income nation".mp. or "low and middle income nation".mp. or (low income countr$ or middle income countr$ or low-income countr$ or middle-income countr$ or low income nation$ or middle income nation$ or low-income nation$ or middle-income nation$).mp. |  |
| 10. (Turkey or Pakistan or Uganda or Sudan or Iran or Lebanon or Bangladesh or Ethiopia or Jordan or Colombia or Brazil or South Sudan or Kenya or Congo or Bangladesh or Peru or (Afghan$ or Iranian$ or Sudan$ or Eritrea$ or Somali$ or Iraq$ or Palestin$ or Venuzual$ Myanm$ or Burm$ or DR of the Congo or DRC or Central African Republic or Burundi or Rohingya or Western Sahara or Hondura$ or Nigeria$ or El Salvador$ or Yemen$ or Syria$ or Kurd$)).mp. |  |
| 11. 1 or 2 |  |
| 12. 3 or 4 or 5 or 6 |  |
| 13. 9 or 10 |  |
| 14. 7 and 8 and 11 and 12 and 13 |  |

**Web of Science – 517**

|  | TS=("asylum seek*" OR refugee* OR migrant* OR immigrant* OR "forcibly displaced" OR "irregular migrants" OR "undocumented migrants" OR "forced migrant" OR "externally displaced" OR "refugee camp") |
| --- | --- |
|  | TS=("human migration" OR "emigration and immigration")  TS=("mental health" OR ((mental* OR psychologic* OR psychiatr*) NEAR/2 (health OR disorder* OR disease* OR deficien* OR illness* OR problem*)))  TS=(flourish* OR "positive psychology" OR "health promotion" OR "positive mental health" OR "subjective well-being" OR "subjective well being" OR "subjective wellbeing" OR "mental well-being" OR "mental well being" OR "mental wellbeing" OR resilience)   \|  \| TS=("Depressive Disorder" or depression or dysthymic disorder or "anxiety disorder" or "panic disorder" or anxiety or agoraphobia or separation anxiety or neurosis or psychoneurosis or "obsessive-compulsive" or "obsessive compulsive" or PTSD or "post-traumatic stress disorder" or "post traumatic stress disorder" or "posttraumatic stress disorder") \| \| --- \| --- \|  \|  \| TS=("cross-cultural comparison" OR "cultural characteristics" OR "cultural diversity" OR "ethnology" OR "Qualitative research" OR "qualitative" OR "ethnological research" OR "cultural adaptation" OR "crosscultural adaptation" OR "cross-cultural adaptation" OR ("purpos*" NEAR/4 "sampl*") OR ("focus" NEAR/1 "group*") OR "observational method*" OR "interview" OR "content analysis" OR (("discourse*" OR "discurs*") NEAR/3 "analys*s") OR "narrative analys*s" OR ("grounded" NEAR/1 ("theor*" OR "analys*s")) OR "action research" OR "account" OR "accounts" OR "unstructured" OR "openended" OR "open ended" OR "narrative*" OR "process evaluation" OR "feasibility" OR "acceptability") \| \| --- \| --- \|  \|  \| TS=("Internet-Based Intervention" OR service* OR delivery OR provision OR model OR programme OR program OR treat OR therapy OR psychother* OR psychosocial OR therapy OR intervent* OR counsel OR support* OR intervention) \| \| --- \| --- \|   TS=("Global Health" OR "Developing Countries" OR "developing countr*" OR "developing countr*" OR "developing nation*" OR “low- and middle-income countr*" OR "low and middle income countr*" OR "low- and middle-income nation" OR "low and middle income nation" OR "low income countr*" OR "middle income countr*" OR "low-income countr*" OR "middle-income countr*" OR "low income nation*" OR "middle income nation*" OR "low-income nation*" OR "middle-income nation*")  TS=(Turkey OR Pakistan OR Uganda OR Sudan OR Iran OR Lebanon OR Bangladesh OR Ethiopia OR Jordan OR Colombia OR Brazil OR "South Sudan" OR Kenya OR Congo OR Bangladesh OR Peru OR Afghan* OR Iranian* OR Sudan* OR Eritrea* OR Somali* OR Iraq* OR Palestin* OR "Venuzual*" OR "Myanm*" OR Burm* OR "DR of the Congo" OR DRC OR "Central African Republic" OR Burundi OR Rohingya OR "Western Sahara" OR Hondura* OR Nigeria* OR "El Salvador*" OR Yemen* OR Syria* OR Kurd*)  #1 OR #2  #3 OR #4 OR #5  #8 OR #9  #6 AND #7 AND #10 AND #11 AND #12 |

**African Journals Online – 180**

**Searched via Google**: site:ajol.info ("asylum seekers" OR "refugee" OR "forced migrant" OR "forced migrants" OR "irregular migrant" OR “immigrants”) AND ("intervention" OR "interventions" OR "therapy" OR "psychosocial" OR "service") AND ("cultural adaptation" OR "acceptability" OR "process evaluation" OR "qualitative" OR "feasibility") AND ("mental health" OR "mental disorder" OR "well-being" OR "depression" OR "anxiety" OR "PTSD")

|  | **VHL Library – 164 (filtering out MEDLINE results)**  TI: ("asylum seeker" OR “asylum seekers” OR refugee OR refugees OR migrant OR migration OR migrants OR immigrant OR immigrants OR "forcibly displaced" OR "irregular migrants" OR “irregular migrant” OR "undocumented migrants" OR “undocumented migrant” OR "forced migrant" OR “forced migrants” OR "externally displaced" OR "refugee camp") |
| --- | --- |
|  | AND TW: (mental health OR mental disorder OR psychological health OR psychological disorder OR psychiatric health OR psychiatric disorder OR mental illness OR psychological illness OR psychiatric health OR flourishing OR "positive psychology" OR "health promotion" OR "positive mental health" OR "subjective well-being" OR "subjective well being" OR "subjective wellbeing" OR "mental well-being" OR "mental well being" OR "mental wellbeing" OR resilience OR "Depressive Disorder" OR depression OR dysthymic disorder OR "anxiety disorder" OR "panic disorder" OR anxiety OR agoraphobia OR separation anxiety OR neurosis OR psychoneurosis OR "obsessive-compulsive" OR "obsessive compulsive" OR PTSD OR "post-traumatic stress disorder" OR "post traumatic stress disorder" OR "posttraumatic stress disorder") AND   \|  \| TW: ("cross-cultural comparison" OR "cultural characteristics" OR "cultural diversity" OR "ethnology" OR "Qualitative research" OR "qualitative" OR "ethnological research" OR "cultural adaptation" OR "crosscultural adaptation" OR "cross-cultural adaptation" OR “purposive sample” OR “focus group” OR “focus groups” OR "observational method" OR “observational methods” OR "interview" OR "content analysis" OR "discourse analysis" OR "discursive analysis" OR "narrative analysis" OR "process evaluation" OR "feasibility" OR "acceptability") AND \| \| --- \| --- \|  \|  \| TW: ("Internet-Based Intervention" OR service OR services OR provision OR programme OR program OR treatment OR therapy OR psychotherapy OR psychosocial OR therapy OR intervention OR interventions OR counsel OR support) AND \| \| --- \| --- \|   TW: (Turkey OR Pakistan OR Uganda OR Sudan OR Iran OR Lebanon OR Bangladesh OR Ethiopia OR Jordan OR Colombia OR Brazil OR "South Sudan" OR Kenya OR Congo OR Bangladesh OR Peru OR Afghan OR Iranian OR Iranians OR Sudanese OR Eritrean OR Eritreans OR Somalian OR Somalians OR Iraqi OR Iraqis OR Iraq OR Palestinian OR Palestinians OR "Venezuelan" OR Venezuelan OR "Burmese" OR “Myanmar” OR "DR of the Congo" OR DRC OR Congolese OR "Central African Republic" OR Burundi OR Rohingya OR "Western Saharan" OR Honduran OR Hondurans OR Nigerian OR Nigerians OR "El Salvadorian" OR “El Salvadorians” OR Yemeni OR Yemenis OR Syrian OR Syrians OR Kurdish) |

**Intervention - 93**

("asylum seekers" OR "refugee" OR "forced migrant" OR "forced migrants" OR "irregular migrant") AND ("intervention" OR "interventions" OR "therapy" OR "psychosocial" OR "service") AND ("cultural adaptation" OR "acceptability" OR "process evaluation" OR "qualitative" OR "feasibility") AND ("mental health" OR "mental disorder" OR "well-being" OR "depression" OR "anxiety" OR "PTSD")

**Open Grey – 9**

("asylum seekers" OR "refugee" OR "forced migrant" OR "forced migrants" OR "irregular migrant") AND ("intervention" OR "interventions" OR "therapy" OR "psychosocial" OR "service") AND ("cultural adaptation" OR "acceptability" OR "process evaluation" OR "qualitative" OR "feasibility")

**ANALAP – 30**

Filtered by: Evaluation reports resource type, health tag, refugees/IDPs tag, (“migrants") AND (intervention) AND ("mental health")

**Total search results:** 2,912

**Search results after Endnote duplication removal:** 2,147

**Search results after Rayaan duplication removal:** 2,055

**NGO Websites Searched**

| http://evaluation.msf.org/reports/evaluation?f[0]=sm_global_local_issues%3A1201 |
| --- |
| https://www.ifrc.org/en/publications-and-reports/evaluations |
| https://publications.iom.int/search-books |
| https://www.islamic-relief.org/evaluations/ |
| https://www.unhcr.org/search |
| https://policy-practice.oxfam.org.uk/publications/search |
| https://www.muslimaid.org/media-centre/news/addressing-the-global-refugee-crisis/ |
| https://resourcecentre.savethechildren.net/keyword/mental-health-and-psychosocial-support |
| https://www.odi.org/search/site/Mental%20health%20refugees |
| https://www.intersos.org/en/ |
| https://doctorswithafrica.org/en/field-research/maternal-child-health/page/5/ |
| https://www.actionagainsthunger.org/impact/nutrition |
| https://en.emergency.it/what-we-do/humanitarian-programmes/ |
| https://www.rescue.org/reports-and-resources |
| https://www.unicef.org/evaldatabase/index_23807.html |
| https://www.unrwa.org/resources/reports?field_program_target_id=39&field_country__target_id=All |
| https://www.caritas.org/who-we-are/publications-and-resources/ |
| https://mhpss.net |
| https://awdf.org/resources/page/5/ |
| https://europe.mercycorps.org/ |
| http://www.volint.it/vis/ |
| www.psicologinelmondo.org |
| https://www.unponteper.it |
| https://apps.who.int/iris/discover |
