# Supplementary material for: How does context influence the delivery of mental health interventions for asylum seekers and refugees in low- and middle-income countries? A qualitative systematic review
Source: Int J Ment Health Syst. 2021 Oct 26;15:80. doi: 10.1186/s13033-021-00501-y (PMC8546788; doi:10.1186/s13033-021-00501-y)
Supplement: Supplementary file 2 — Additional file 2: Appendix S2. Quality Appraisal. [file 13033_2021_501_MOESM2_ESM.docx]

**Quality Appraisal using the Critical Appraisal Skills Programme (CASP, 2018)**

|  | **Aladro et al, 2020** | **Brown et al, 2020** | **Burchert et al, 2019** | **Chapman et al, 2019** | **Greene et al, 2019** | **Gürle et al, 2019** | **Hakki et al, 2020** | **Makhoul et al, 2011** | **Murray et al, 2018** |
| --- | --- | --- | --- | --- | --- | --- | --- | --- | --- |
| **1.Was there a clear statement of the aims of the research?** | **✔️** | **✔️** | **✔️** | **✔️** | **✔️** | **✔️** | **✔️** | **✔️** | **✔️** |
| **2.Is a qualitative methodology appropriate?** | **✔️** | **✔️** | **✔️** | **✔️** | **✔️** | **✔️** | **✔️** | **✔️** | **✔️** |
| **3.Was the research design appropriate to address the aims of the research?** | **✔️** | **✖️** | **✔️** | **✖️** | **✔️** | **✔️** | **✔️** | **✖️** | **✔️** |
| **4.Was the recruitment strategy appropriate to the aims of the research?** | **✔️** | **✖️** | **✔️** | **✔️** | **✔️** | **✔️** | **✔️** | **✔️** | **✔️** |
| **5. Was the data collected in a way that addressed the research issue?** | **✔️** | **✖️** | **✔️** | **✔️** | **✖️** | **✖️** | **✔️** | **✖️** | **✔️** |
| **6. Has the relationship between researcher and participants been adequately considered?** | **✔️** | **✖️** | **✖️** | **✖️** | **✔️** | **✖️** | **✔️** | **✔️** | **✖️** |
| **7.Have ethical issues been taken into consideration?** | **✔️** | **✖️** | **✔️** | **✖️** | **✔️** | **✖️** | **✖️** | **✖️** | **✖️** |
| **8. Was the data analysis sufficiently rigorous?** | **✔️** | **✖️** | **✔️** | **✖️** | **✔️** | **✖️** | **✖️** | **✔️** | **✔️** |
| **9. Is there a clear statement of findings?** | **✔️** | **✔️** | **✔️** | **✔️** | **✔️** | **✔️** | **✔️** | **✔️** | **✔️** |

|  | **Nakkash et al, 2019** | **Rebolledo et al, 2019** | **Sim et al, 2018** | **Sullivan et al, 2019** | **Tay et al, 2019** | **Tol et al, 2011** | **Vijayakumar et al, 2017** | **Yassin et al, 2018** | **ZaghroutHodali et al, 2019** |
| --- | --- | --- | --- | --- | --- | --- | --- | --- | --- |
| **1.Was there a clear statement of the aims of the research?** | **✔️** | **✔️** | **✔️** | **✔️** | **✔️** | **✔️** | **✔️** | **✔️** | **✔️** |
| **2.Is a qualitative methodology appropriate?** | **✔️** | **✔️** | **✔️** | **✔️** | **✔️** | **✔️** | **✔️** | **✔️** | **✔️** |
| **3.Was the research design appropriate to address the aims of the research?** | **✔️** | **✔️** | **✔️** | **✖️** | **✔️** | **✖️** | **✔️** | **✔️** | **✔️** |
| **4.Was the recruitment strategy appropriate to the aims of the research?** | **✔️** | **✖️** | **✔️** | **✖️** | **✖️** | **✔️** | **✔️** | **✔️** | **✖️** |
| **5. Was the data collected in a way that addressed the research issue?** | **✔️** | **✔️** | **✔️** | **✖️** | **✖️** | **✔️** | **✔️** | **✔️** | **✖️** |
| **6. Has the relationship between researcher and participants been adequately considered?** | **✔️** | **✖️** | **✔️** | **✖️** | **✖️** | **✔️** | **✔️** | **✔️** | **✖️** |
| **7.Have ethical issues been taken into consideration?** | **✖️** | **✖️** | **✔️** | **✖️** | **✖️** | **✔️** | **✔️** | **✔️** | **❓** |
| **8. Was the data analysis sufficiently rigorous?** | **✖️** | **✖️** | **✖️** | **✖️** | **✔️** | **✔️** | **❓** | **✔️** | **✖️** |
| **9. Is there a clear statement of findings?** | **✔️** | **✔️** | **✔️** | **✔️** | **✔️** | **✔️** | **✔️** | **✔️** | **✖️** |
